# The Role of Collective Action in Enhancing Communities’ Adaptive Capacity to Environmental Risk: An Exploration of Two Case Studies from Asia

**DOI:** 10.1371/currents.RRN1279

**Published:** 2011-10-24

**Authors:** Philip Ireland, Frank Thomalla

**Affiliations:** ^*^Macquarie University, Department of Environment and Geography, Australia and ^†^Department of Environment and Geography, Faculty of Science, Macquarie University

## Abstract

Background

In this paper we examine the role of collective action in assisting rural communities to cope with and adapt to environmental risks in Nepalgunj, Nepal and Krabi Province, Thailand. Drawing upon two case studies, we explore the role of collective action in building adaptive capacity, paying particular attention to the role of social networks.

Methods

Data for this paper was gathered using a range of different methods across the two different studies. In Nepal semi-structured interviews were conducted with a range of stakeholders in addition to participant observation and secondary data collection. In Thailand the researchers utilised a vulnerability assessment, participatory multi-stakeholder assessment, a detailed case study and an online dialogue.

Findings

We make three key observations: firstly, collective action plays a significant role in enhancing adaptive capacity and hence should be more strongly considered in the development of climate change adaptation strategies; secondly, social networks are a particularly important component of collective action for the building of adaptive capacity; and thirdly, the mandate, capacity, and structure of local government agencies can influence the effectiveness of collective action, both positively and negatively.

Conclusions

We argue that there is an urgent need for further consideration of the different forms of collective action within community-based disaster risk management and climate change adaptation.

## **1. Introduction**

As the concern over dangerous climate change impacts has intensified in recent years there has been a growing focus on adaptation in both developing and developed countries. A significant number of development institutions, including Multilateral Development Banks, bilateral agencies and International Non-Government Organisations (NGOs) are engaging in this emerging field. Climate change presents a wide range of significant new challenges for communities across the world including rising sea levels, changing weather patterns and a greater intensity and frequency of climate related hazards.  Early approaches to climate change adaptation have drawn upon a range of existing strategies [1] [2], particularly Disaster Risk Reduction (DRR) [3] [4]. It has been widely recognised by theorists and practitioners alike that many pre-existing approaches to preparing for and coping with environmental risks are likely to be useful in enhancing adaptive capacity to climate change [5] [2] [6]. In this paper we draw upon two different case studies from Asia to explore the potential role of collective action at the community level in enhancing adaptive capacity to environmental changes and risks, including climate change.    

The concept of collective action has long been a consideration in development theory and practice [7] [8] [9] and has also emerged in discussions on climate change adaptation [1]. Definitions of collective action generally assert that it involves a group of people that voluntarily engage in a common action to pursue a shared interest [10] [11]. It can take the form of resource mobilisation, activity coordination, information sharing or the development of institutions (Poteete and Ostrom (2004). Poteete and Ostrom (p.216) [8] call for more research that identifies the factors that facilitate or hinder collective action. In relation to climate change, Adger [1] argues that collective action is at the core of adaptation decisions related to the management of resources associated with agriculture, forestry and other resource dependant livelihoods. Whilst it is evident that collective action can act as an enabler for climate change adaptation [1] [6] its precise role in enhancing adaptive capacity in communities remains unclear.      

Adaptive capacity is one of the key concepts within the field of climate change adaptation. The IPCC defines adaptive capacity as the “ability of a system to adjust to climate change (including climate variability and extremes) to moderate potential damages, to take advantage of opportunities, or to cope with the consequences” (p.72) [12]. Within the literature adaptive capacity has been primarily examined through the concepts of thresholds and coping ranges [13]. Smit and Wandel frame these terms as “conditions that a system can deal with, accommodate, adapt to and recover from” (p. 287)[14]. Other theorists have explored which factors enhance or reduce adaptive capacity such as social and physical drivers [15] and governance systems [6]. Whilst Adger agues that collective action may play a role in enhancing adaptive capacity he calls for more case specific research at various scales (p. 400)[1]. Recent research on this topic includes Toni and Holanda’s [16] study of the effect of land tenure on droughts in north-eastern Brazil. They found that farmers involved in common property pasturelands as opposed to private pasturelands, were on average less vulnerable to climate variations due to more diversified management and husbandry systems. Similarly Jodha [17] found that the integration of mountain communities from the Himalayas into the mainstream market economy, away from a collectivised one, has negatively affected traditional adaptive capacity to climate extremes. For example integration had contributed to the disappearance of indigenous knowledge systems and collective risk sharing arrangements that had been “safeguarding against vulnerability” (p. 36)[17]. In this paper we employ two different case studies from Asia to further explore how collective action affects adaptive capacity in these communities. In our analysis we pay particular attention to the role of social networks and the interaction of collective action with local governance.   

Social networks are defined as links or relationships between households, communities and institutions of governance that facilitate the flow of material and non-material resources [18] [19]. Only limited research has been conducted on the role of social networks in the enhancement of adaptive capacity to climate change. For example, Tompkins and Adger [20] explored co-management of coastal resources in Trinidad and Tobago to suggest that collective action opens up new lines of community communication and facilitates increased influence on government. They argue that this can enhance the adaptive capacity of the community by increasing the number of resources that are available to communities [20]. Similarly, Ensor and Berger [21] suggest that local social networks can offer marginalised groups an opportunity to develop adaptive strategies. In our exploration of social networks we also draw upon Adger’s [1] theories on bonding and networking social capital (p. 389). Social capital refers to the key components social organisation, such as trust norms and networks, which facilitate collective action and enhance economic performance [22] [23]. According to Adger [1], bonding social capital refers to relationships of kinship and friendship whereas networking social capital pertains to relationships beyond the immediate group and can involve actors at different levels in the community, such as government.   

## **2. Methods**

We draw on data collected in two different Asian case studies on collective action in the context of environmental changes and risks. The first case study is concerned with the establishment and functioning of women’s collectives in the rural town of Nepalganj in Nepal. Data for this case study was gathered during doctoral research of the first author. This included five weeks of field research in Nepal during 2010 and interviews conducted with Australian NGO employees [24] [25] who had been involved with the women’s collectives during 2009. Semi-structured interviews were utilised as the primary method and were conducted with eleven local and international development actors including NGO employees, local government representatives and members of women’s collectives. Due to the relatively small size of Nepalganj, these participants were selected based upon their involvement with the women's collectives. Semi-structured interviews enabled a flexible exchange that sought to privilege the participants voice. This approach is consistent with post-colonial methodology whereby knowledge construction can operate as a two way low of learning. Other methods that were utilised were participant observation, field diary and secondary data collection.

The second case study focuses on community based disaster risk management and early warning system development in Krabi Province, Thailand. Insights for this case study were derived from several projects the second author has been involved in between 2005 and 2009 that related to the sustainable recovery and resilience building after the 2004 Indian Ocean Tsunami. This research included a vulnerability assessment of Thai tourist destination communities [26]; a participatory multi-stakeholder assessment of early warning system - community linkages [27]; a detailed case study of community disaster preparedness and early warning in Krabi Province, Thailand [28]; and a global Online Dialogue on Early Warning [29].    

Key similarities between the two case studies are that they both focus on rural communities, both are located in Asia, and both are current examples of communities faced with environmental risks. A key difference is that the case study from Thailand is concerned with coastal hazards (particularly tsunamis) whereas the case study from Nepal focuses weather related hazards such as flash flooding and droughts. They hence address different environmental risks. However, while a tsunami is not a consequence of climate change, its impacts along the coast can be compared to the impacts of other coastal hazards that are related to climate change, such as tropical cyclones and severe storms. Another difference is that the case studies are located in different countries with different languages, cultures, religions and governance systems. By focusing on these two case studies we are able to undertake a comparative analysis of factors enhancing and hindering collective action and to make broader observations that are likely to be relevant outside the specific situations and contexts of these communities. All the research that is presented in this paper was approved by the relevant institutions ethics processes and committees.  

## **3. Women’s collectives in Nepalganj, Nepal**

### ***3.1 The context of the collectives***

We now consider the first case study that involves a group of women’s collectives that engage in a range of activities aimed at reducing vulnerability to a range of social and environmental risks. The collectives are located near the town of Nepalganj in the Banke district of the Terai of Nepal in the Karnali Basin approximately 8km from the Indian boarder. Over the past 15 years, Nepalganj has experienced considerable socio-economic changes including rapid urbanisation and population increases. These are to a large extent the result of regional conflicts and changing work migration patterns [25]. The majority of the participants of these women’s collectives are low caste urban poor women without a regular income. Nepalganj’s climate is controlled by the Asian monsoon. In this region climate change is expected to impact the Asian monsoon by increasing interannual variability [12]. This in turn is likely to manifest in decreased rainfall during the winter, an unpredictable start date to the monsoon rains, and less frequent and more intense monsoon rainfall [12].   

The women’s collectives in Nepalganj were initially formed through the facilitation of a local NGO. Between 1998 and 2005, female community members from certain sections of the town of Nepalganj were invited by a Nepali NGO to participate in a group action process. This participatory development method encouraged the women to think about the challenges they faced and potential solutions. The women identified many issues relating to the weather and climate, particularly the impacts of floods and droughts on their livelihoods. For example, in recent years there has been less agricultural work available due failed crops and the late onset of the monsoon and related cultivation processes. The result of this group process was the formation of collectives that supported a range of activities aimed at reducing vulnerability to these environmental risks. These include: savings and loans schemes that made available finance as a buffer in times of hardship such as drought, collective agriculture initiatives that provided food to the group members and collective business enterprises, such as a mushroom farm, that provided additional streams of income. All these actions increased the resources that the group members could draw upon in general, and during times of environmental shocks and surprises.  

### ***3.2 Functioning of the collectives***

The collectives functioned initially with a relatively uniform operational structure. The facilitators were employed by a local NGO and they led the initial formation and functioning of the groups. Each collective consisted of 6 to 35 members. Over time the collectives elected a leader and an assistant leader from within their group. These leaders would then be responsible for organising the collective in their geographical area. The NGO facilitator would serve as a resource and guide for the group with a gradually diminishing presence. The facilitators would attend group meetings for the first few years and assist by taking minutes and developing the skills of other group members. As the number of collectives increased, regional committees were established, in coordination with the facilitating NGO, and these were attended and run by the leaders of the local collectives. These committees provided additional coordination and links between the groups and increased their capacity to engage issues on a different scale, such as regional health care, and facilitate larger projects such as raised cement roadways. The regional committees provided an additional level of social network that enhanced networking social capital.    

One of the main functions of the collectives was to establish and manage a credit and saving scheme. This capital available for loans was raised and managed by the members themselves. The NGO acted as a facilitator of the process rather than a source of credit. Meetings were held regularly to administer loans and repayments. Money for the group was raised by its members in the form of savings with a low interest rate. This could be loaned by individuals or drawn upon for the purpose of group projects when necessary. Through a consensus decision making system groups autonomously decided on the priorities for expenditure of these funds. Examples of this include the financing of labor for the installation of donated toilets and loans to those in the group who needed the assistance most urgently [24]. As the collectives became more established they increasingly began to lobby local government representatives to provide better services to the community.   

### ***3.3 Contribution to adaptive capacity***

This case study illustrates four important contributions of collective action to the strengthening of adaptive capacity. First, collective action facilitated the establishment and strengthening of social networks. These networks serve as communication channels for new knowledge relating to environmental changes and risks, planning processes, and emergency information during times of environmental stress and hazards. One development actor involved in the project stated: “*the development of new relationships and networks among the participants was just as valuable as the practical, or tangible, outcomes of the project*” . Given the uncertainties of climate predictions and the range of potential surprises faced by communities, networks that can rapidly disseminate new and updated information are crucial in enabling effective adaptation responses. Secondly, collective action improved the economic resources of the members of the women’s collectives. By increasing the resources of the participants, the vulnerability of the participating individuals and communities was reduced. A savings scheme created a financial reserve that individuals could draw on in times of hardship. In terms of climate risks, these funds were sometimes used to help people after a crop had failed due to adverse weather conditions. The capacity to adapt to uncertain environmental risks was also enhanced by the micro-credit and savings scheme. The administration, ownership and management of this scheme was organised by the collectives. The joint operation of these saving schemes by the collective is likely to further strengthen social networks.  

Collective action can also provide a space for community members to voice, discuss and solve problems. This is the third contribution of collective action to adaptive capacity we identify from this case study. The group action process facilitated the identification of both the problems and solutions. The ability to downscale modeled climate change projections to regional and local scales remains limited to-date but in many cases adaptation responses will nevertheless need to be identified and implemented by local actors. The development of more robust decision making frameworks enhances the capacity of local actors to adapt to a range of environmental changes and risks. Finally, collective action contributes to individual and collective empowerment that can establish and strengthen relationships with local government actors and lead to stronger advocacy. Many of the members of the women’s collectives noted that they and their families felt more confident in the community as a result of their participation in the collectives. Because of this empowerment members of the collectives felt more confident to meet with local government officials and to request better community services, such as improved access to clean drinking water and the cleaning of drains. This empowerment is a manifestation of what Adger [1] defines as the networking capital form of collective action.   

### ***3.4 Interaction of women’s collectives with local governance processes***

In general people in the community, and members of the collectives, did not trust the government due to widespread and long-term issues of corruption. An NGO actor noted “*it is generally understood by Nepalis that government is corrupt and filled with nepotism*”. This situation has been exacerbated by the absence of local elections in recent years due to regional conflicts and instability in the national government. However, despite these issues we identified several notable examples of positive interaction between the collectives and local government actors.   

Being a part of a collective enabled their members to engage in advocacy with their local government. All members of the collectives were part of the marginalised section of the community as a result of a range of intersecting factors such as landlessness, gender and unemployment. The government engaged in only a very limited way with this demographic for a range of cultural and social reasons, including the legacy of the caste system and differing religious backgrounds. The members of the collectives expressed that on their own they felt voiceless and that they were initially reluctant to approach the government. Through the initial group action process many of the collectives decided that some of the challenges they were facing should be addressed by the government. For example, inadequate and blocked public drains frequently resulted in local flooding during monsoonal rains. Many of the collectives hence started to lobby the government to provide better services.  Members of the collectives reported that they felt more confident to talk to their local government officials to request funds and services as a result of the collective process. After several years these actions resulted in a range of tangible positive outcomes including improved drainage systems, roads and the provision of additional land for agriculture. Several members reported that as their sense of empowerment increased, their perception of themselves changed and they became bolder. Collective action challenged the experience of disconnection between communities and local government.     

### ***3.5 Enabling and constraining factors of collective action***

We now identify enabling and constraining factors for collective action in Nepalganj. Factors that enabled the continuing functioning and growth of collectives included a long-term commitment from the local NGO, the capacity of key actors, and the development of social networks. The long-term support of the local NGO provided secure employment to the local facilitators and enabled the program to develop and improve methods over time. Hancock [25] argues that the level of commitment of facilitators and group leaders provided a long-term and committed engagement with the communities that ultimately precipitated the formation of the collectives. Many participants observed that the development of social relationships between members of the collectives and to other collectives both strengthened the continuing functioning and the formation of new collectives [24] [25] The enhancement of social networks in the community enabled the collectives to include more participants and to help a larger number of people. Some of the collectives also extended their services to men.   

A range of constraining factors limited the success of the collectives. These mostly pertain to culture, religion, politics, governance, capacity, and resource availability. In some cases, different religious and political views and affiliations hampered the relationships between collectives and the wider community and local government actors. For example, Worboys [25] reports that some government representatives refused to acknowledge or meet with some collectives because their members were affiliated with a different religion or a different political party. A patriarchal system within the society provided an impediment for some women being able to participate in the collectives.  Gender also created a barrier for some women in trying to meet with male government staff. Members of some collectives reported that certain government representatives refused to meet with them unless they were accompanied by men.  One local government employee reported that he had lost social status by interacting with the women collective (p. 40)[25]. Finally, an overall lack of available resources, both individually and from local government, provided a consistent challenge for many of the collectives. In addition to scarce financial resources Hancock [24] noted that low levels of literacy sometimes created difficulty in recording savings and loans and day-to-day challenges such as sickness and poor access to healthcare reduced attendance at meetings.   

## **4. Community-Based Disaster Risk Management in Krabi Province, Thailand**

### ***4.1 The context of the collectives***

Krabi is one of the southern provinces of Thailand and is located along the Andaman Sea. The Andaman Coast was the most severely affected area of Thailand by the 2004 Indian Ocean Tsunami. The tsunami devastated the provinces of Phang-Nga, Krabi, Phuket, Trang, Satun, and Ranong [30]. In Krabi Province, Phi Phi Island was the worst damaged, and, in particular, the main tourist areas of Ton Sai Bay and Loh Dalum Bay [31]. At least 2,000 people are presumed dead [32] [33] and 15,812 people were directly affected [30]. Following the 2004 tsunami, community-based disaster risk management activities in Krabi Province were initiated by a range of international, national and local organisations, and by the communities themselves. Much of the work of international and national NGOs and government authorities has so far focused on communities that were devastated by the tsunami, and some communities that are perceived as highly vulnerable to future events.   

### **4.2 *Functioning of the collective action***

Community-based disaster risk management is frequently organised through disaster preparedness committees in which people act as unpaid volunteers for the greater good of the community. For example, the Thai arm of CARE International, the Raks Thai Foundation stated that it is important to implement projects in communities that are already organised [27]. The idea to work in organised communities relates to the notion that it is useful to recognise and build on existing strengths within communities and to work with people that are already actively engaged at the local level. For this reason and because it is difficult to initiate and sustain a new committee for disaster risk management, many NGOs work with existing committees, such as funeral or loan committees. These kinds of committees exist to support members with small loans during times of hardship.   

Motivations for participation in a disaster preparedness committee are many and varied. In many coastal communities a principal source of motivation for investing in such a committee is a high awareness of coastal hazards due to the high loss of life during the 2004 tsunami. Another motivation is strongly linked to failures of formal governance responses at the local and a lack of trust in the commitment and capability of government authorities to provide effective disaster risk management. For example, in the tourism communities of Krabi Province emergency aid relief did not reach all eligible recipients; funding was insufficient and available funds were often misappropriated due to corruption and nepotism at the local level [34]. Tensions between communities and local government authorities exist also because of other unresolved issues, such as the use of illegal fishing gear and practices in some communities. Concerns over livelihoods are another important driving force for engaging in community-based disaster preparedness activities. Many communities do not have an interest in disaster risk management per se, but are willing to engage in these activities if they also lead to livelihood improvement.  

Collective actions focus on the enhancement of disaster awareness and preparedness of community members and tourists, capacity building for disaster risk management and early warning, and the mobilisation of support from local government and NGOs. Disaster preparedness activities include the collection and dissemination of existing information; the identification of hazards, potential impacts, high-risk areas, safe areas, evacuation routes and those most vulnerable to hazards; the development of public awareness campaigns and school programmes; the preparation of emergency plans; and early warning and evacuation exercises. Activities aimed at capacity building include the recruitment and training of volunteers in emergency response activities such as search and rescue and first aid skills and the development of alternative warning dissemination infrastructure and procedures.   

Support for community-based collective action is mobilised in different ways. One mechanism is established social networks with community-based organisations (CBOs) in neighbouring communities. Another mechanism that is becoming increasingly popular with some NGOs is micro-credit schemes. For example, the Raks Thai Foundation uses a Revolving Loan Fund^2^ as an initial entry point for engaging with communities.    

Important examples of collective action in Thailand include the “Mister Tuan-Pai" (“Mr Early Warning”) project at the national level, the Save the Andaman Network (SAN) at the sub-national regional level and the “One Tambon One Search and Rescue Team (OTOS) at the local level. The "Mister Tuan-Pai" project was established under the Disaster Prevention and Mitigation Office (PDPMO) to recruit volunteers to monitor flash flood hazards using rainfall gauges and to provide early warnings to villages in areas at risk. SAN is an informal network of NGOs and CBOs that was established in response to a perceived lack of coordination by the international NGOs in post-tsunami recovery. OTOS was established by the Department of Disaster Prevention and Mitigation (DDPM), the Department of Local Administration, the Health Insurance Office, the Office of Health Promotion and Support Fund, and the Thai Red Cross to 1) ensure the safety of life, and rapid and efficient search and rescue operations; 2) establish efficient search and rescue teams at every Province, District and Tambon (Sub-district) in the country; 3) enhance the capacity and efficiency of search and rescue teams through technical training and drills; 4) build up the self-confidence of search and rescue teams; and 5) provide first aid treatment and rapid transfer to the appropriate medical establishment. OTOS is under the administration of the Local Administration Organisation, responsible for controlling traffic during evacuations.  

### ***4.3 Contribution to adaptive capacity***

The insights derived from the participatory assessment of early warning system – community linkages [27] show that stakeholder agency and collective action are important elements of the adaptive capacity of communities. Building capacity to cope with environmental (as well as socio-economic) shocks and surprises is an important step towards adapting to climate change. An important aspect of adaptive capacity is what people can do to help themselves through collective action.    

The evidence from Krabi Province suggests that local government agencies in some cases lack the capacity to support collective action and can therefore represent a considerable barrier. This is an important governance issue that is also relevant in the context of adaptation projects. Recently, progress has been made in two areas: the first one is a transition in the approach of many governments and NGOs from a focus on post-disaster emergency response to addressing longer-term development that links disaster risk reduction with livelihoods, natural resource management and poverty reduction efforts. The second is the integration of disaster risk reduction and climate change adaptation. This is a subject of considerable current debate but a number of synergies have been put forward by various authors (see e.g., [2] [3] [4]). We argue that in addition to these two integrative steps, adaptive capacity could be enhanced by better defining and coordinating the roles and responsibilities of government, NGO and private sector actors because this would remove tensions, competition and the duplication of efforts.     

In the online dialogue on early warning [29] responses on the theme ‘technology versus community’ indicated that efforts to strengthen disaster risk reduction and early warning are heavily biased towards technology. There is an urgent need to go beyond such technological approaches and to recognise the importance of investing in the capacities of communities by developing and better utilising social networks. The role of social networks has not been recognised sufficiently in the current debate despite it’s potential as an important part of future disaster risk reduction and climate change adaptation strategies. While response capability depends largely on the community’s own capacity to manage risks, the engagement of government and NGO actors to inspire and support collective action is crucial.   

This case study demonstrates that community-based action is positioned in the context of multiple needs and interests and that there is a challenge in coordinating multiple stakeholder agendas. Community-based disaster risk management, therefore, needs to be integrated in strategies that address wider community priorities, such as improving and diversifying livelihoods and building capacity for community-based natural resource management. Addressing these concerns helps to build adaptive capacity to climate change because the underlying causes of vulnerability to shocks and surprises are reduced.   

### ***4.4 Interactions with local governance processes and other organisations***

In many cases, there is a lack of financial and staff capacity for disaster risk management activities at the lower levels of government. Many stakeholders shared a general concern over a lack of human resources, knowledge, experience and skills relating to disaster risk management and a lack of government initiative from sub-national authorities. Contributing to the lack of capacities at sub-national levels are high staff turn-over and a lack of political will to engage in disaster risk management due to many other responsibilities and the prioritisation of other issues that are considered more important. There is also a lack of trust amongst the public in government institutions due to poor public services provision and to corruption. In Khao Lak and Phi Phi Island Calgaro et al.  found that “emergency aid relief did not reach all eligible recipients” and the “available funds were often misappropriated due to corruption and nepotism operating at the local level” (p. 47)[34]. Many communities therefore have very little trust in the government’s commitment and capacity to develop effective disaster risk reduction strategies.   

Additional challenges exist in the collaboration and communication between government and other stakeholders, such as universities, NGOs and CBOs and the private sector. Local government representatives are not trained to facilitate processes to engage with communities. NGOs tend to be much better at communicating with communities and have well-established methods. However, some local government authorities are reluctant to facilitate NGO initiatives in communities because they don’t want to relinquish authority to the NGOs. Despite this, NGOs often play an important role in supporting communities to initiate planning for community-based disaster risk management, to engage with the local government, to access information and guidance, and to receive financial support.   

### ***4.5 Key enabling and constraining factors***

There were a number of factors that enabled collective action.  Our research demonstrates that strong leaders with good social networks are an important enabling factor for community-based disaster risk management. Leaders include those who may not have a formal position but who nevertheless have influence in their communities [29]. For example, in the village of Koh Panyee, the Rescue Team draws on experience from the village health committee that was already well established. The chairman of the health committee has a key role in the Rescue Team, because he is perceived as competent due to his university education and computer skills [35]. Religious leaders can play an important role in building disaster preparedness because they can disseminate information on hazards and disaster preparedness initiatives in their services.   

Many young people volunteer their time to these activities because of incentives such as free services such as health care, training in language and other skills that are beneficial for seeking employment and advancing careers. Volunteering also enhances social status and supports political advancement through local electorates in Tambon Administrative Organisations. Participation and ownership of procedures and early warning systems by the community is also an important enabling factor.    

Another enabling factor is the integration of disaster risk management into strategies that address wider community priorities, such as improving and diversifying livelihoods and building capacity for community-based natural resource management. In our experience in Krabi Province, the ‘framing’ of activities is crucial in the process of partnership building and bringing people onboard, where the approach has to be expressed in terms that are relevant for the partners.  

We observed a range of barriers to collective action on disaster risk management in Krabi Province. Because of limited resources and capacities of sub-national government actors, the different priorities and lack of political will of some local authorities, and insufficient coordination between local government and NGO actors [28] [34], local government frequently represent a barrier to collective action.    

While community leaders often play a critical role in enabling and facilitating action within their communities they can also hamper collective action. For example, some village leaders show no interest in disaster risk management despite interest of the community and this can create conflict and may lead to isolation from NGO and government activities. In Thailand, the village headman plays an important role because he is elected as a representative of the central government and he is elected for life. The political context at the community level is therefore strongly shaped by the politics at the national level and there have been cases of bribery and allocation of funds to relatives of the headman.  

A lack of resources at the local level negatively affects the ability of committees to act and to induce positive change in their communities. Volunteers also often lack authority. For example, many young volunteers don’t feel comfortable telling older people what to do and many older people do not take them seriously. Many CBOs have limited legal status and are not recognised by government authorities as legitimate stakeholders in disaster risk reduction and early warning system planning and implementation processes. Whilst there is an increasing emphasis on participatory planning , participatory practices have not yet been mainstreamed into humanitarian action[36] [37].  

In some communities affected by the 2004 tsunami, the importance of disaster preparedness is not fully understood because there is no history of disasters. The Thai philosophy of life “Mai Pen Rai” (English equivalent “Not to worry” or “Never mind”) could be interpreted as complacency. In Muslim society, religious and cultural beliefs about predetermined destiny (fatalism) are often difficult to overcome.    

Some government authorities are concerned about the negative image disaster preparedness activities might shed on tourism communities as safe and pristine destinations. The value of community-based disaster risk management and community empowerment is contested; sometimes it is difficult to convince people that investing in disaster risk management is as important as investing in livelihoods and that activities might create co-benefits for both.    

Despite the dedication of volunteers, there is concern about the longer-term sustainability of disaster preparedness efforts that rely to a large extent on volunteerism because even volunteers require basic financial support for operational logistics such as transport, food, and compensation for the loss of income. The high turnover of volunteers and the need to continuously recruit and train new people puts a considerable strain on organisational capacities.   

Guidance for community-based disaster risk management is not always available to communities or directly useful in the local environmental and socio-economic context of a particular community. In Ban Tha Klong, Thailand, the village committee described that the government provides information and seminars on tsunamis, landslides, and sea-level rise but that there was a lack of access to information on natural resource management and experience, good practice and technical guidance on disaster risk reduction. Several villages committees told us that in order to plan disaster risk management activities, they require detailed information about the community, including infrastructure, population distribution and density, location of vulnerable social groups, geographical maps of the terrain, disaster areas, and tourism areas. These data are usually held by government authorities and requests from village committees to obtain such information are not always successful. However, one could question whether such data-driven planning is useful for local action.

## **5. Discussion**

The case studies presented in this paper build upon the literature to explore how collective action enhances adaptive capacity. Whilst both are context specific and deal with different issues they both involve collective action in response to environmental changes and risks amongst rural communities in developing countries. It is groups such as these that are particularly vulnerable to climate change and are hence in need of local level adaptation responses. In this section we provide an overview of the case studies and investigate two emerging themes. We consider the role of social networks as a critical component of collective action that enhances adaptive capacity and explore the impact of government institutions in supporting and hampering collective action.   

Table 1 - Summary table of key attributes of collective action in the two case studies

**Table:** 

	**Women’s collectives **	**Disaster committees **
**What was the context? **	A poor and marginalised section of the community in a rural town in Nepal	Rural coastal communities in Krabi Province, Thailand, affected by 2004 tsunami
**How did the collective action emerge? **	NGO facilitated the formation and initial running of self-help women’s collectives	Initiated by a wide range of international, national and local organisations, and the communities themselves; frequently built on existing committees
**What was the motivation?**	The NGO had a mandate for development and the women involved sought to better their material context	Concern over future coastal hazards, perceived lack of action of the government, and concerns over livelihood security
**What were the activities of the collective action?**	-Group savings -Budgeting -Sustainable business loans, -Advocacy to government, -Organisation for group projects	-Enhancement of disaster preparedness of the community -Capacity building for disaster risk reduction and early warning, -Mobilisation of support from local government and NGOs
**Contribution to adaptive capacity**	-enhances social networks for information dissemination -improves access to finances -provides a space to discuss and address new issues, such as climate change -facilitates individual empowerment through group action	-addresses underlying causes of vulnerability -builds capacity to cope with shocks -links disaster risk reduction with livelihoods, natural resource management, poverty reduction -integrates disaster risk reduction and climate change adaptation. -helps to better define and coordinate different actors
**Relationship with local government **	-Widespread mistrust amongst the community of government -Significant issues around mandate, structure and capacity -Established collectives lobbied their local councilors with relative levels of success	-Lack of trust amongst communities in government institutions -Lack of human resources, knowledge, experience and skills relating to disaster risk reduction and a lack of government initiative from sub-national authorities, -Challenges in the collaboration and communication between government and other stakeholders
**What were the constraining factors**	-Gender conflicts -Cultural and religious conflicts -Lack of resources at all levels -Political and social disempowerment -context of poverty	-Lack of experience and awareness of hazards. -Religious and cultural beliefs about destiny. -Lack of support from political and religious leaders. -Lack of resources. -Lack of information and guidance.
**What are the enabling factors **	-The commitment of individual actors, -Long-term funding commitment from international donors -Good staffing -Robust social networks	-Incentives that are beneficial for employment -Existing social networks -Support from political and religious leaders. -Integration with other priorities.

The critical importance of social networks to the process of adaptation to climate change is supported through our case study observations. In both case studies the development of social networks was an important factor in enhancing adaptive capacity. In Nepalganj the women’s collectives provided a platform that enabled and facilitated social networking. Utilising Adger’s [1] ideas, the collectives both enhanced bonding capital within the community and cultivated networking capital with local government actors through the process of advocacy. However, it should be noted that it is difficult to differentiate between these two forms of social capital as the relationships are rarely characterised by one category but instead exist in a complex web of interactions. For example, in Nepalganj an advocacy relationship with a government representative subsequently developed into a friendship. In this instance the process of acting collectively cultivated enhanced social networks. Similarly, in the context of the disaster preparedness committees, social networks are a crucial feature of collective action. The importance of collective action and the associated social networking is recognised by one of the participants of the Online Dialogue for Early Warning: “*community organisation is more important than investing in high-tech solutions*” (see also [29]). Thomalla et al [27] argue that effective disaster risk management depends upon strong personalities and good social networks. Adger notes that “*the social dynamics of adaptive capacity are defined by the ability to act collectively*” (p. 396)[1].   

The case studies presented here also contribute new insights into our understanding of the characteristics that enable or hinder collective action at the community level.  We observe that social networks and collective action remains largely informal in nature - it is not integrated into and therefore not supported by the formal governance system. Larsen et al. [38] argue that this occurs despite the considerable progress made in institutionalising international and national formal governance structures for disaster risk reduction in the public sector.  The findings contrast with the observations of Adger [1] and Ensor and Berger [21]that a community’s network produces an open and productive relationship with government. Agrawal [39] and Pototee and Ostrom [8] emphasise the importance of local institutions in both the cultivation and long-term effectiveness of collective action.  In examining the interactions between community action groups and local government we find that local government actors, rather than enabling collective action, tended to hamper the ability of communities to realise their full potential. On the other hand, collective action can play an important role in strengthening local government and making it more accountable through increased scrutiny.   

Local government may constrain collective action and hence inhibit adaptation to climate change.  The most notable issues hampering collective action are corruption and nepotism, a lack of financial and staff capacity, ineffective and poorly-coordinated governance structure, diverging priorities and a lack of political will of local leaders, strained relations with NGOs and communities, and correspondingly considerable distrust of communities amongst government officials. In both case studies, stakeholders emphasise the importance of strong and motivated individuals with good social networks who take leadership of the common cause. A comment that represents the view was made by a development practitioner in Nepalganj who stated: “*leaders play a critical role in enabling or constraining [community action]*”.   

The case studies show that collective action is not always compatible with the local governance system. In Krabi Province, one research participant noted that “*collective action is outside the scope and experience of local government*”; another stated “*government doesn’t have the capacity to facilitate collective action because they have not been trained with the skills*” (p. 12) [35]. Many current approaches to disaster risk reduction continue to lack integration with sub-national and national governance structures. Progress in coordination and integration of different government, NGO and community actors needs to be made if the various levels of government are going to be tasked with the development and implementation of disaster risk reduction and adaptation strategies.  

## **6. Conclusion**

The evidence presented in this paper indicates that collective action has a significant role to play in enhancing the adaptive capacity of communities to environmental changes and risks, including climate change. This both reinforces and builds upon the claims made by Adger [1], Eakin et al. [6] and Johda [17]. Further, this paper shows that social networks are an important component of collective action that contributes to adaptive capacity. Finally, the case studies demonstrate that a more robust nexus of relationships occurs if collective action builds upon existing social networks and is supported by formal governance structures and processes.    

This research highlights the importance of local government – community interactions in supporting or hampering collective action. Having competent and committed individuals in local governance processes that have access to adequate human and financial resources is a strong enabler of effective action at the community level. Equally important are the role and influence of certain interest groups, and the existing barriers due to limited resources and capacities, different priorities and approaches, distrust and tensions, and a lack of coordination of local government, NGO and private sector actors.  

Despite its demonstrated importance in reducing vulnerability and building adaptive capacity to environmental changes and risks, we have shown that collective action occurs largely within informal social networks and governance structures. These informal governance structures, supported by communities and NGOs, frequently exist in parallel with formal governance structures. There is an apparent need to reconcile informal and formal governance because the current disconnect causes tensions and conflicts between different stakeholders engaged in disaster risk reduction and climate change adaptation. These conflicts hamper the establishment of ethically acceptable processes in which the underlying vulnerabilities of communities can be effectively addressed.  This tension between formal and informal governance raises the question of how we recognise and support the building of adaptive capacity that occurs in informal spheres.    

The relationships between local government actors and local collectives also warrant further investigation: More research needs to be undertaken to understand how institutions can best support community-based adaptation. Do the principles of collective action contradict the culture and assumptions of contemporary governance? Brooks suggests that it is the vested political and economic relationships that “*determine the nature of the adaptation context*” (p. 12)[40]. Similarly, Ensor and Berger identify issues of governance and empowerment as key and state: “*these political and institutional challenges are at the heart of community based adaptation*” (p. 6)[21]. We agree with Moser’s [41] argument that we need a greater understanding of the opportunities, barriers and limits to adaptation through a critical analyses of the socio-economic and political power dynamics that underpin vulnerability and adaptive capacity. Because collective action has an important role to play in enhancing the adaptive capacity of communities, we must carefully consider the potential interactions of community-led adaptation strategies with existing governance structures in order to strengthen community efforts and to avoid ineffective programs and wasted resources.    **Acknowledgements**

For the case study on Nepal we would like to acknowledge Mr Russell Hancock for his assistance with this research and the time and energy he has put into supporting collective action in Nepal. The research presented in the case study on community-based disaster risk management is based on work undertaken by the Stockholm Environment Institute (SEI), Macquarie University, Sydney, and partners in the Indian Ocean Region with financial support from the Swedish International Development Cooperation Agency (Sida). We acknowledge the contributions of Mr Rasmus Klocker Larsen at SEI and Dr Emma Calgaro at Macquarie University to the stakeholder consultations in Thailand. All relevant project reports are referenced throughout the paper. Finally, we would like to thank Dr Louis Lebel of Chiang Mai University, Thailand, for his comments on an earlier draft of the paper. 

**Funding Information**

The research presented in the case study on community-based disaster risk management is based on work undertaken by the Stockholm Environment Institute (SEI), Macquarie University, Sydney, and partners in the Indian Ocean Region with financial support from the Swedish International Development Cooperation Agency (Sida). The research presented on the case study in Nepal was funded by Macquarie University.

**Competing interests**

The authors have declared that no competing interests exist.
